# Does cardiopulmonary bypass increase the risk of postoperative acute kidney injury after coronary artery bypass grafting?

**DOI:** 10.1186/cc14373

**Published:** 2015-03-16

**Authors:** A Karmali, C Walker, L Kuppurao

**Affiliations:** 1 Harefield Hospital, London, UK

## Introduction

Acute kidney injury (AKI) following coronary artery bypass grafting (CABG) results in significant morbidity, mortality and prolonged stay on the cardiothoracic ICU. We sought to determine the incidence of AKI in our cardiothoracic centre, hypothesizing a higher occurrence in patients undergoing CABG using cardiopulmonary bypass (ONCAB) compared with off-pump (OPCAB) surgery [1].

## Methods

Retrospective data from all nondialysed patients undergoing isolated CABG at our institution were collected for the year 2013. Propensity scoring using the software platform MatchIt® was used to match subjects from ONCAB and OPCAB groups with regard to preoperative variables: logistic EuroSCORE, creatinine clearance (CrCl), gender and operative urgency. Postoperative AKI was defined as a rise of 50% or more in baseline serum creatinine [2]. Chi-square analysis was used to determine statistically significant differences.

## Results

From 500 cases (369 OPCAB, 131 ONCAB), 262 subjects were included in the final analysis, with 131 in each group. There was a higher incidence of AKI and renal replacement therapy (RRT) in the ONCAB group, although this was not significantly greater than in the OPCAB group (Table 1). The mortality rate was identical with three deaths in each group. The average length of ICU stay for the OPCAB group was 1.96 days versus 2.49 days for the ONCAB group.

**Table 1:** 

	OPCAB	ONCAB	*P *value
AKI	14 (10.69%)	19 (14.50%)	0.35
RRT	6 (4.58%)	9 (6.87%)	0.43

## Conclusion

Our study is limited by its size and the use of logistic EuroSCORE as a composite measure of risk factors. However, it has demonstrated that in our predominantly off-pump cardiac unit, OPCAB conferred no statistically significant advantage over ONCAB with regard to postoperative AKI. Studies to date have standardised patient-specific variables, but not the conduct of the procedure itself; for example, minimising renal hypoperfusion during OPCAB. This may be a reason for the lack of clear superiority in the continuing debate over choice of CABG procedure and reducing the risk of AKI.
